# Cell membrane inspired nano-shell enabling long-acting Glucose Oxidase for Melanoma starvation therapy via microneedles-based percutaneous delivery

**DOI:** 10.7150/thno.60758

**Published:** 2021-07-13

**Authors:** Yang Zeng, Haiyan Zhou, Jinsong Ding, Wenhu Zhou

**Affiliations:** 1 Xiangya School of Pharmaceutical Sciences, Central South University, Changsha, Hunan, 410013, China; 2 Department of Pathology, School of basic medicine, Central South University, Changsha, Hunan, 410013, China

**Keywords:** Skin cancer, starvation therapy, catalysis, metal organic frameworks, polydopamine

## Abstract

**Rationale:** Glucose oxidase (GOx) has gained tremendous research interest recently as a glucose-consuming enzyme for tumor starvation therapy, while its *in vivo* applications are strictly limited by rapid deactivation, as well as side effects of non-specific catalysis.

**Methods:** To address these issues, here we report a protective nano-shell to encapsule GOx for localized melanoma therapy delivered by dissolving microneedles (MNs). Inspired by cell membrane that separates and protects cell organelles and components from outside environment while selectively ingesting nutrition sources, we designed polydopamine (PDA)-structured nano-shell to allow free transportation of glucose for catalytic reaction, while impede the penetration of GOx, proteinase, and other GOx-deactivating macromolecules across the shell membrane.

**Results:** GOx was well protected in core layer with persistent catalytic activity for at least 6 d under various biological matrixes (*e.g.*, PBS, serum, and cell lysate) and surviving different harsh conditions (*e.g.*, acid/base treatments, and proteinase-induced degradation). Such long-acting nano-catalyst can be easily integrated into MNs as topical delivery carrier for effective glucose consumption in melanoma tissue, achieving significant tumor growth inhibition via starvation therapy with minimized side effects as compared to systemic administration.

**Conclusion:** This work provides an elegant platform for *in vivo* delivery of GOx, and our cell-mimicking nano-system can also be applied for other enzyme-based therapeutics.

## Introduction

Metabolic pathways are important therapeutic targets for tumor therapy 1-3. Abnormally proliferative cancer cells need abundant nutrients and energy supply to support their survival and growth 4, 5. To meet rapid energy requirement, cancer cells predominantly produce energy through glycolysis even in presence of abundant oxygen, which is known as Warburg effect, making cancer cells highly sensitive to the fluctuation of glucose concentration 6, 7. Enlightened by this fact, various tumor starvation therapies have been proposed to deplete the intratumoral glucose, such as glucose transporter inhibitors to circumvent glucose metabolism 8, 9, vascular embolization to cut off nutrient supply 10, and glucose oxidase (GOx) to directly consume glucose 11, 12. Glucose oxidase (GOx) catalyzes the oxidization of glucose into gluconic acid and hydrogen peroxide (H_2_O_2_), which has gained increasing interest in the biomedical field recently 13-17. By virtue of its catalytic oxidation of glucose without energy production, GOx is a highly efficient glucose depletor for tumor starvation therapy. Notably, the byproducts of gluconic acid and H_2_O_2_ could enhance the acidity of the tumor microenvironment and provide substrate for other treatment methods, enabling the integration of multi-modal tumor therapies for synergistic effect. For instance, GOx has been combined with other enzymes 18, hypoxia-activated prodrugs 11, photosensitizers 19, or Fenton reagents 20, for cooperatively enhanced tumor therapy.

However, GOx-based tumor therapy is still in its infancy, and there exist significant problems for its potential clinical applications. Since the substrate of glucose and oxygen are ubiquitous in body, non-specific GOx catalysis could inevitably cause unwanted side effects, such as glycopenia, tissue hypoxia, and reactive oxygen species (ROS) generation 21. Moreover, GOx can be easily degraded and deactivated by proteinase during *in vivo* delivery, thus requiring frequent administration to achieve the desired efficacy 22. Ideally, GOx should be precisely delivered into tumor without any influence on normal tissues, and maintain its catalytic activity for a long period of time for effective intratumor glucose consumption. While various nanoplatforms have been developed for GOx delivery 23-25, none of these systems can achieve all these goals. Therefore, advanced strategies are deemed necessary to drive forward the clinical translation of GOx-based tumor starvation therapy.

Since systemic administration of GOx-loading vehicles may catalytically cause glycopenia, several studies chose intratumoral injection 26-28. While it can minimize non-specific side effects and decrease dosage, this invasive method is clinically unfavorable, and the injected drugs easily leak to neighboring normal tissues, resulting in the loss of effective drug in tumor area as well as potential toxicity 29. On the contrary, microneedles (MNs) are much better choice for topical drug therapy owing to their advantages of non-invasive skin penetration and transdermal drug delivery. MNs are needle arrays with micrometer dimensions that could effectively penetrate the skin barriers for transdermal drug delivery 30. Owing to their easy operation and excellent skin-penetration ability, MNs have been widely employed to deliver various therapeutic drugs 31-34, and the applications of MNs for skin cancer (such as melanoma) therapy has also been explored 35, 36. However, no attempt has been made to percutaneously deliver GOx for localized tumor starvation therapy.

**Scheme 1:**
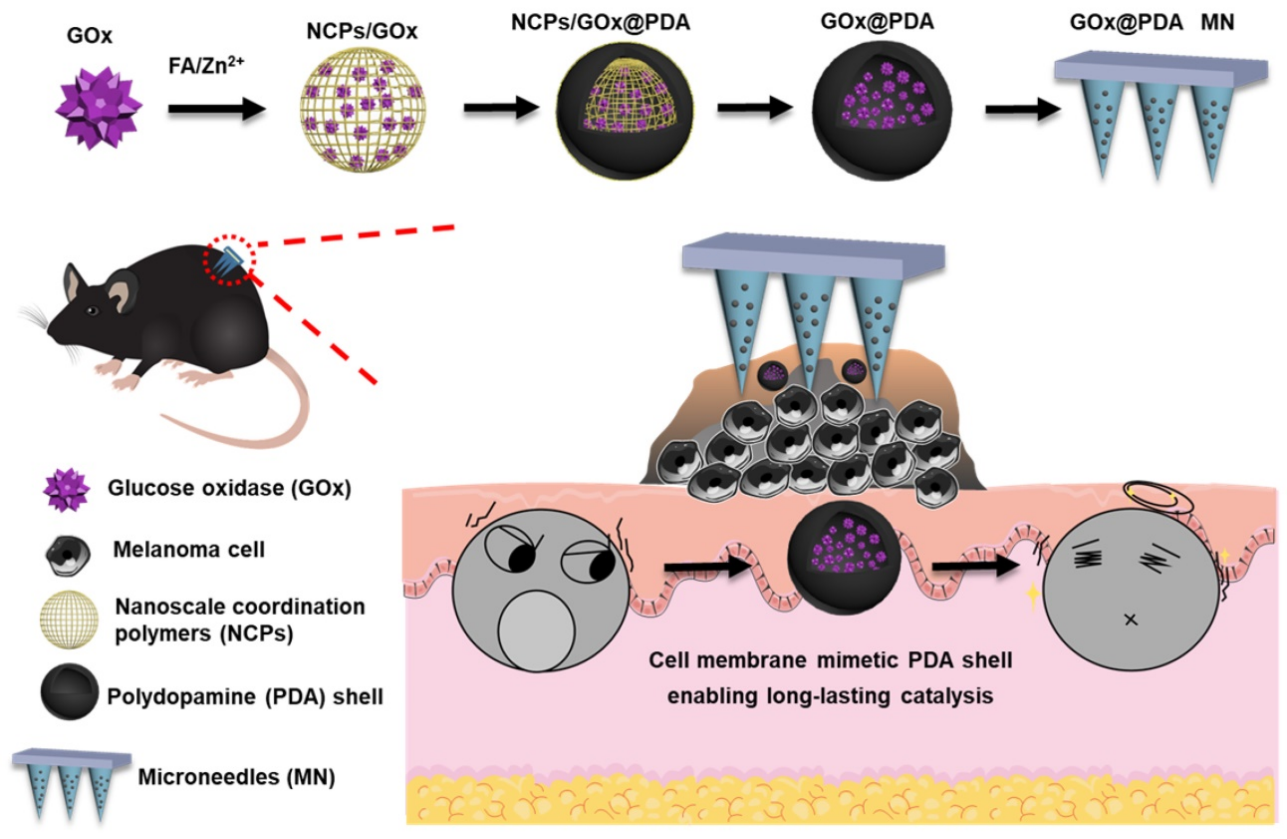
Schematic illustrating the construction of the GOx-loaded PDA nano-shell for long-lasting catalysis, and transdermal delivery via dissolving MNs for percutaneous treatment of melanoma.

Here, we designed and fabricated dissolving MNs to achieve highly efficient topical delivery of GOx for melanoma therapy (Scheme 1). Such MNs could self-dissolve in skin once penetration, and release the payloads without causing adverse effects 37. Importantly, GOx was loaded into a polydopamine (PDA)-based nano-vehicle to enable long-lasting catalytic activity. To prepare this nanosystem, GOx was first biomineralized into nanoscale coordination polymers (NCPs) via coordination between folic acid (FA) and Zn^2+^ ion 38, followed by surface coating a PDA shell layer through self-polymerization of dopamine 39, 40. Upon washing to etch the NCPs core for detoxification, the biocompatible PDA nano-capsule was formed with core loading of GOx. Akin to cell membrane, such PDA shell protected GOx from biological degradation, while allowed penetration of glucose for catalytic reaction, enabling long-lasting catalysis *in vivo*. The MNs were applied in B16F10 tumor-bearing mice model, and robust tumor growth inhibition was achieved with excellent biosafety.

## Results and Discussions

### Characterizations of GOx@PDA

**Figure 1:**
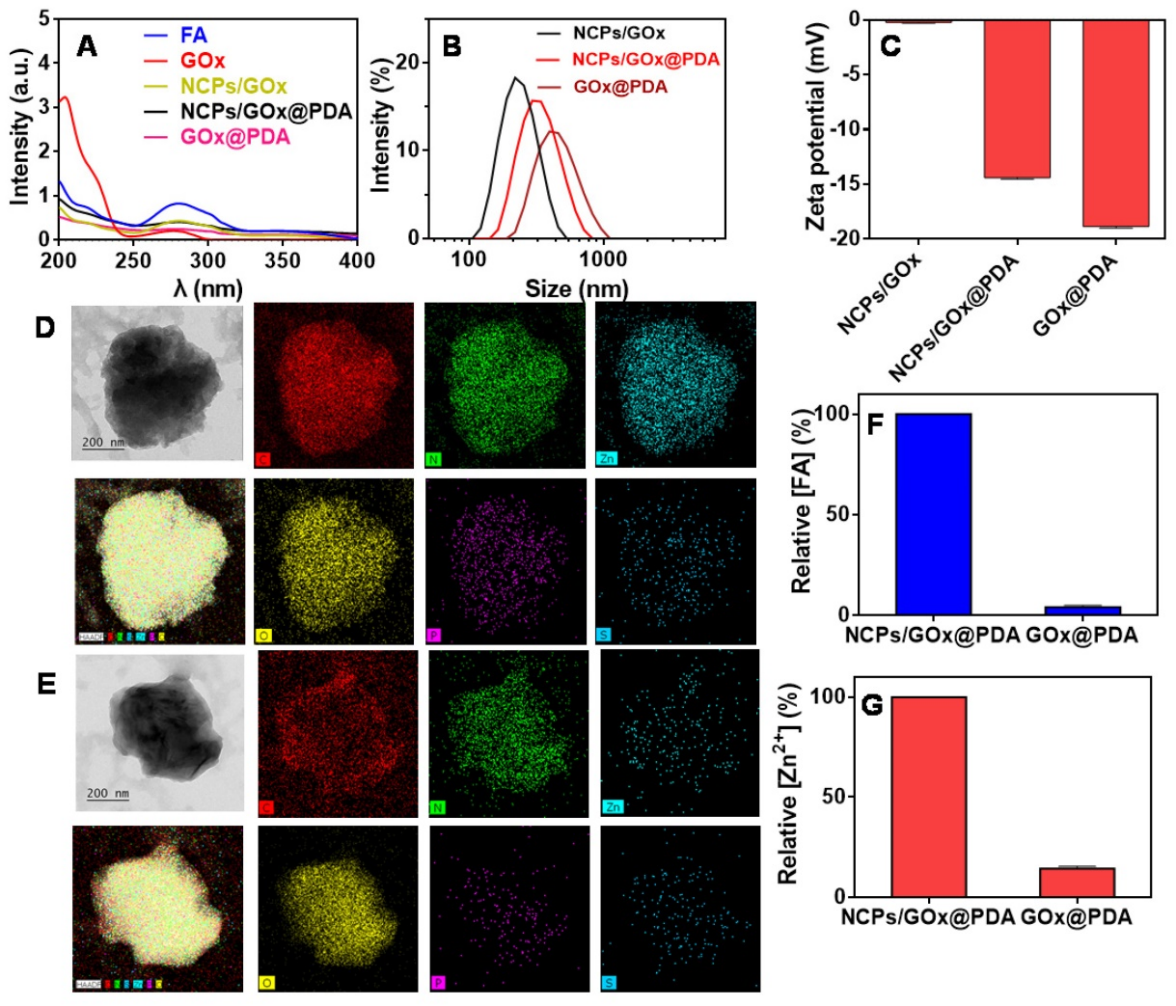
Characterizations of GOx@PDA NPs. (A) UV-Vis spectra of NCPs/GOx, NCPs/GOx@PDA, and GOx@PDA. Hydrodynamic size distribution (B) and zeta potential (C) of NCPs/GOx, NCPs/GOx@PDA, and GOx@PDA. The TEM images and elemental analysis of NCPs/GOx@PDA (D) and GOx@PDA (E). The scale bar: 200 nm. The relative content of (F) FA and (G) Zn^2+^ in NCPs/GOx@PDA and GOx@PDA.

First, the GOx was loaded into NCPs through the coordination between FA and Zn^2+^ (scheme 1), and the successful formation of NCPs/GOx was confirmed by TEM (Figure S1), with GOx encapsulation efficiency (EE%) of 60%. Such effective GOx loading was benefited from the high porous of NCPs, strong coordination of the protein with Zn^2+^ 41, as well as the π-π stacking interactions between the molecules 41-43. Within NCPs/GOx structure, both GOx (275 nm) and FA (280 nm) displayed a red-shift of the UV-vis spectrum, confirming the π-π stacking interactions (Figure 1A) 44. The NCPs/GOx was further coated by a PDA shell layer through the self-polymerization of DA under alkaline condition. After PDA coating, the resulting NCPs/GOx@PDA showed a significant increase of particle size (Figure 1B), as well as the decrease of ζ-potential (Figure 1C). The representative UV-Vis absorbance of GOx was also masked by the dense and thick PDA shell (Figure 1A). We then washed the nanoparticles to remove the NCPs core, and the obtained GOx@PDA showed no obvious change of the particle morphology (Figure 1D-E, Figure S2). From FT-IR spectra, the characteristic peaks of GOx were disappeared in GOx@PDA, likely due to dense PDA encapsulation (Figure S3). The EE% of GOx@PDA was calculated to be 58%, indicating minimal leakage of GOx during etching process. To confirm the nano-capsules structure, the nanoparticles were analyzed by EDS-based elemental mapping (Figure 1D-1E). The C, O, N and Zn elements were densely distributed throughout the NCPs/GOx@PDA nanoparticles, indicating the PDA capsulated coordination structure. Notably, the P and S elements were also observed inside the particle, which was originated from GOx. For GOx@PDA, by contrast, the signal of Zn markedly weakened, due to the dissociation and subsequent leakage of the NCPs upon PBS washing. To quantify this, the amount of FA and Zn^2+^ inside nanoparticles were measured by HPLC and HQS as a fluorescent Zn^2+^ probe, respectively, and both components decreased significantly (Figure 1F-G). For instance, the content of Zn element reduced by ~10-fold through 1 h etching (Figure S4). Therefore, 90% NCPs can be removed after etching, and it is likely to form a yolk-shell structure with dense and thick PDA shell to load GOx. Since the key concern of NCPs as drug delivery carrier is the potential toxicity of the metal ions as well as the organic ligands for *in vivo* applications, our nanocage system has elegantly addressed this issue via removing the whole NCPs core. Then, the colloidal stability of GOx@PDA was studied under different conditions by monitoring the dynamic particle size, while minimal size change was observed after 48 h incubation (Figure S5A), indicating high stability for biological applications. In addition, the long-term stability was also tested, and the nanoparticles can be stored in aqueous solution without an obvious size change for at least 30 d (Figure S5B).

### Long-lasting catalytic activity of GOx@PDA

**Figure 2:**
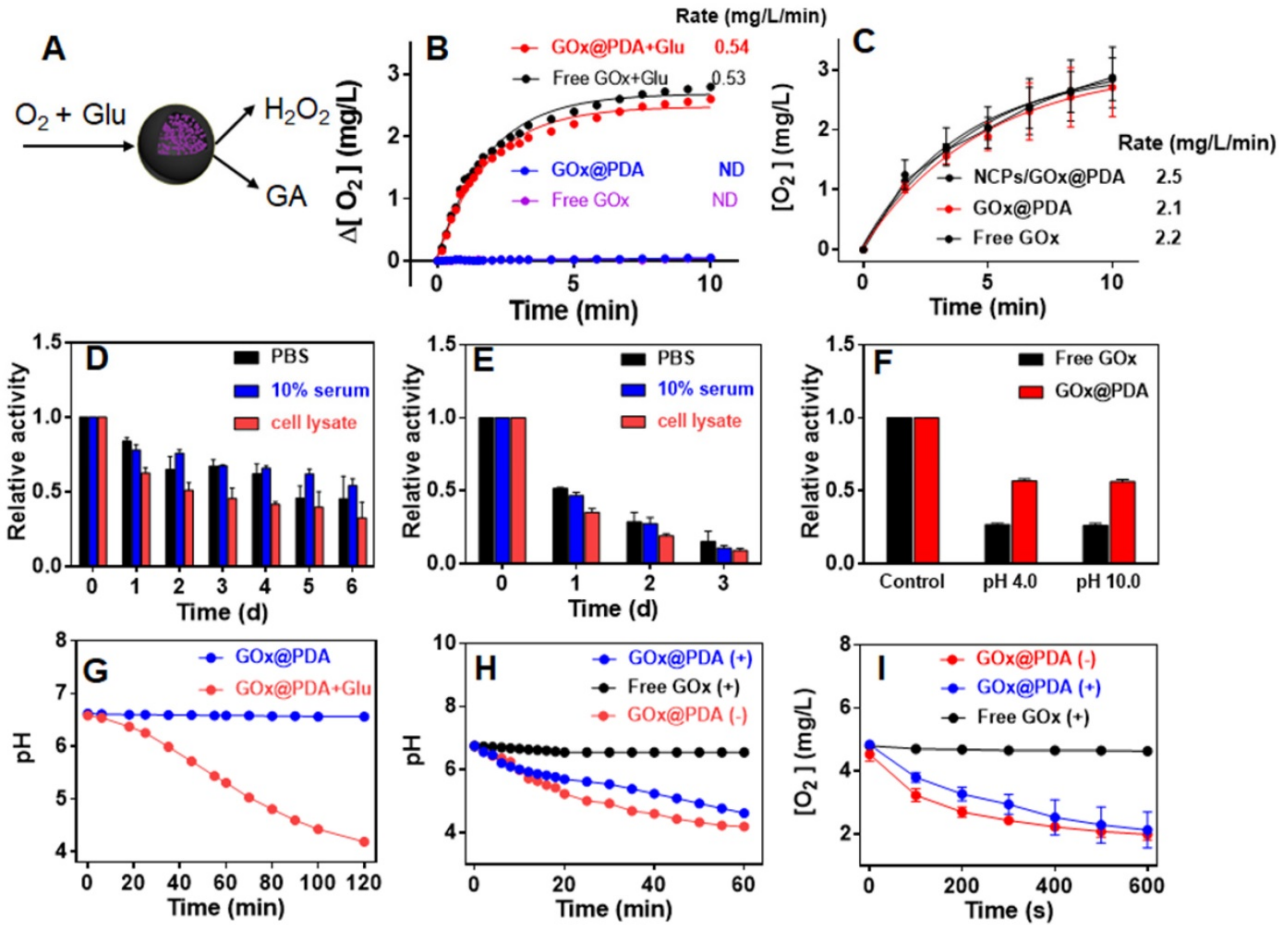
(A) Schematic illustration the catalytic reaction of GOx@PDA. (B) Dynamic monitoring the dissolved oxygen level indicating catalytic activity of GOx@PDA and free GOx ([GOx] = 0.5 mg/mL) in absence or presence of 2.5 mg/mL glucose. ND, no detectable. (C) Dynamic monitoring the dissolved oxygen level indicating the activity of NCPs/GOx@PDA, free GOx and GOx@PDA ([GOx] = 0.167 mg/mL) in presence of 2.5 mg/mL glucose. Relative catalytic activity of (D) GOx@PDA and (E) free GOx after incubation with PBS buffer, 10% serum and cell lysate for different timepoints. (F) Relative activity of GOx@PDA and free GOx after treatments with acidic (pH 4.0)/base (pH 10.0) buffers for 10 min. (G) The activity GOx@PDA as indicated by pH change in absence or presence of 2.5 mg/mL glucose. The activity of GOx@PDA and free GOx after pretreatment with proteinase K (5 mg/mL) for 2 h as measured by the decrease of (H) pH and (I) dissolved oxygen. The symbols of “+” and “-” indicate in presence and absence of proteinase K, respectively.

GOx can oxidize the glucose (Glu) into glucuronic acid (GA) to cut off the supply of energy source, accompanied by the generation of toxic H_2_O_2_ (Figure 2A), which co-contribute to the anti-tumor efficacy. To test the enzymatic activity, the dissolved O_2_ was measured as a probe of the oxidative reaction. Free GOx or GOx@PDA alone has little effect on O_2_ level in solution, while upon addition of Glu the concentration of dissolved O_2_ rapidly decreased from 6.2 to 3.3 (mg/L) in 600 s (Figure 2B), indicating high catalytic efficiency. Based on the oxygen consumption rate, it showed that GOx@PDA achieved quite similar catalytic activity to NCPs/GOx@PDA and free GOx (Figure 2C), indicating minimal GOx loss during PBS washing. Meanwhile, the catalytic activity of GOx was maintained after encapsulation into GOx@PDA.

The key advantage of our system is the PDA capsule to protect GOx from *in vivo* digestion. To explore this protection effect, we challenged the GOx@PDA with various physiological conditions, including PBS buffer, 10% serum, and cell lysate from B16F10 melanoma cells. After different incubation periods, the GOx@PDA was collected and tested for relative activity by examining O_2_ consumption. Among the tested buffer, the cell lysate had the most significant influence on GOx@PDA activity, while it still remained 70% activity after 24 h incubation (Figure S6). For the free GOx, by contrast, less than 30% activity was retained after 24 h incubation with cell lysate. With such notably protection effect, we further expanded the incubation time up to 6 d (Figure 2D). While the activity of GOx@PDA progressively decreased, it still possessed 50% activity in the 10% serum. However, the free GOx was almost completely inactivated after 3 d (Figure 2E). Therefore, our designed nano-capsule system could enable long-lasting activity of GOx for *in vivo* applications. Encouraged by these results, we further challenged the GOx@PDA with various harsh conditions. It is known that proteinases are susceptible to denaturing by acid/base treatments, and as expected, significant activity decrease of free GOx was observed under such treatments (Figure 2F), while GOx@PDA remained much higher activity, demonstrating the versatile protection effect of PDA shell for GOx. Note that under tumor acidic/intracellular endosomal microenvironments (pH 6.5 and 5.5), GOx@PDA could almost maintain its catalytic activity (Figure S7), demonstrating its capability for tumor therapy.

Another way to probe the catalytic activity is to measure pH decrease due to the production of acidic GA, which allows long-term activity monitoring. A gradual decrease of pH was observed for GOx@PDA in presence of Glu over a period of 2 h (Figure 2G). We next tested the proteolytic resistance of GOx@PDA by adding proteinase K. Compared with free GOx that was completed inactivated upon proteinase K treatment, the activity of GOx@PDA was only moderately changed (Figure 2H), demonstrating robust resistance of GOx@PDA from enzymatic digestion. We then challenged the nanosystem with three rounds of proteinase K treatments, while the catalytic activity still remained pretty high (Figure S8), which also demonstrated the multiple turn-over of the catalytic reaction. To confirm this result, the proteinase K-pretreated GOx@PDA was collected for further enzymatic quantification by monitoring the change of dissolved oxygen, and as expected, the catalytic rate was almost unchanged over three times treatments (Figure 2I, Figure S9). For free GOx, by contrast, the activity was completely abolished upon proteinase K pretreatment. We reason that this proteolytic resistance effect was originated from the dense and thick PDA shell layer, which prevents the penetration of both GOx and proteinase K across the capsule membrane while allows free transportation of glucose substrate. To demonstrate this, the release of GOx was also assayed under different physiological conditions, and GOx was merely released over 24 h (Figure S10), confirming the stable encapsulation.

### *In Vitro* Antitumor Assay

**Figure 3:**
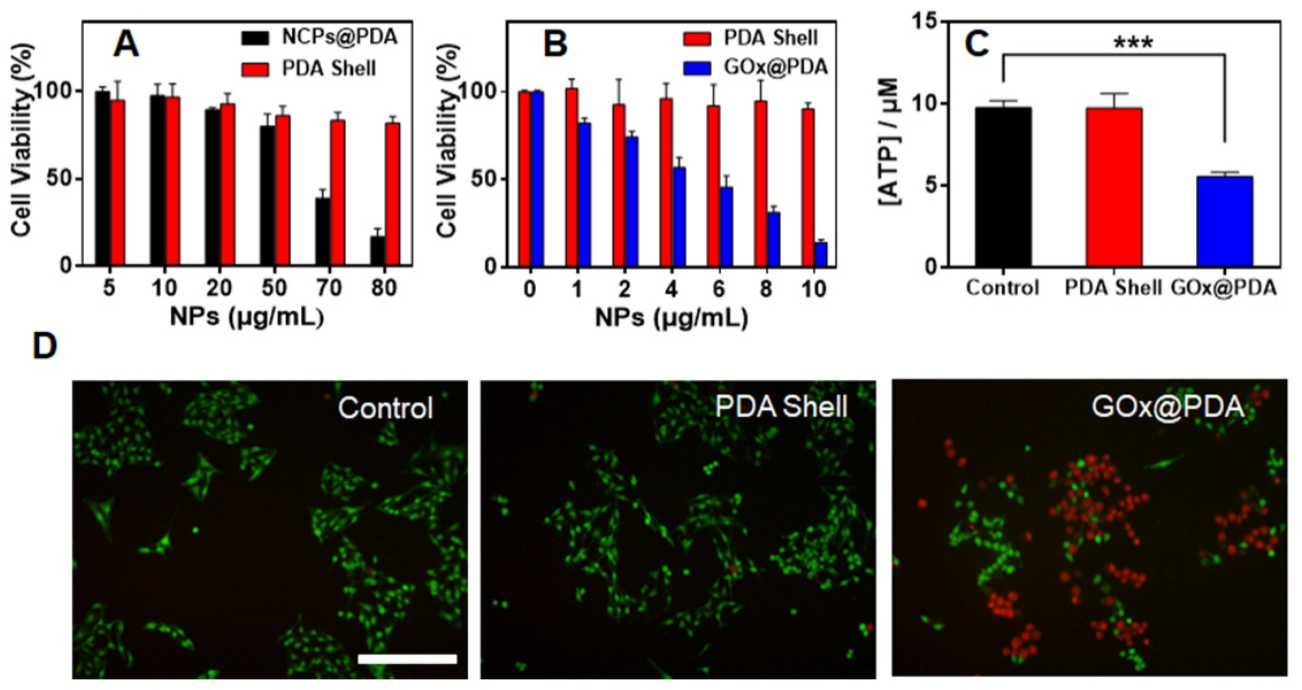
(A) Cell viability of B16F10 cells after treatment with PDA shell and blank NCPs@PDA at different concentrations. (B) Cell activity of B16F10 cells after treatment of PDA shell and GOx@PDA at different concentrations for 48 h incubation. (C) ATP level in B16F10 cells after treatments with PBS, PDA and GOx@PDA. (D) Representative live/dead cell images of B16F10 cells after different treatments. Scale bar: 100 μm.

After systematic characterization, we next studied *in vitro* therapeutic efficacy by MTT assay using B16F10 cells melanoma cells as example. As control experiment, the cytotoxicity of the nanocarriers were evaluated (Figure 3A). Without GOx loading, the blank NCPs@PDA showed strong cytotoxicity at high concentration, which is likely due to the release of Zn^2+^ that generates reactive oxygen species (ROS) to damage cells 45. Upon washing to remove the NCPs core, by contrast, the PDA shell displayed quick high biocompatibility with >80% cells viable after treatment. Therefore, the NCPs@PDA nanocarrier can be effectively detoxified upon removing the NCPs core, which further verifies the superiority of PDA nano-capsules for GOx delivery. Further, the cytotoxicity of GOx@PDA was explored, and a concentration-dependent cell damage was observed (Figure 3B), attributable to the consumption of nutrient glucose for starvation therapy. Compared to free GOx, GOx@PDA achieved significantly enhanced anti-tumor efficacy (Figure S11), which can be ascribed to the protective effect of the PDA shell. To confirm this mechanism, intracellular adenosine triphosphate (ATP), the main carrier of energy, was measured after treatment. The ATP level significantly reduced for GOx@PDA group due to catalytic glucose depletion, while the no change was observed for the PDA shell (Figure 3C). Note that catalytic generation of H_2_O_2_ could also damage cells 46. To explore this, the H_2_O_2_ generation was monitored by using DCFH-DA probe that can emit green fluorescence in response of H_2_O_2_. The cells with PDA shell treatment did not show any fluorescent signal, while bright fluorescence was observed inside cells after GOx@PDA treatment (Figure S12), indicating H_2_O_2_ production. Therefore, catalytic generation of H_2_O_2_ may also contribute to the anti-tumor effect of GOx@PDA.

For convenient observation, the cytostatic activity was further evaluated by live/dead cell fluorescence co-staining. Compared with the untreated control, the PDA shell group showed bright green fluorescence inside cells, indicating high viability of the cells (Figure 3D). Upon GOx@PDA treatment, by contrast, intensified red fluorescence was observed, suggesting high cell damage effect. This result was highly consistent with the above MTT data. To test the generality, we applied the nanosystem to various types of cancer cells, all of which showed high response, resulting in IC_50_ values of 4.74-6.29 μg/mL (Figure S13). Overall, the GOx@PDA displayed excellent anti-tumor activity against various types cancers, showing great potential for subsequent *in vivo* applications.

### GOx@PDA loaded MNs

**Figure 4:**
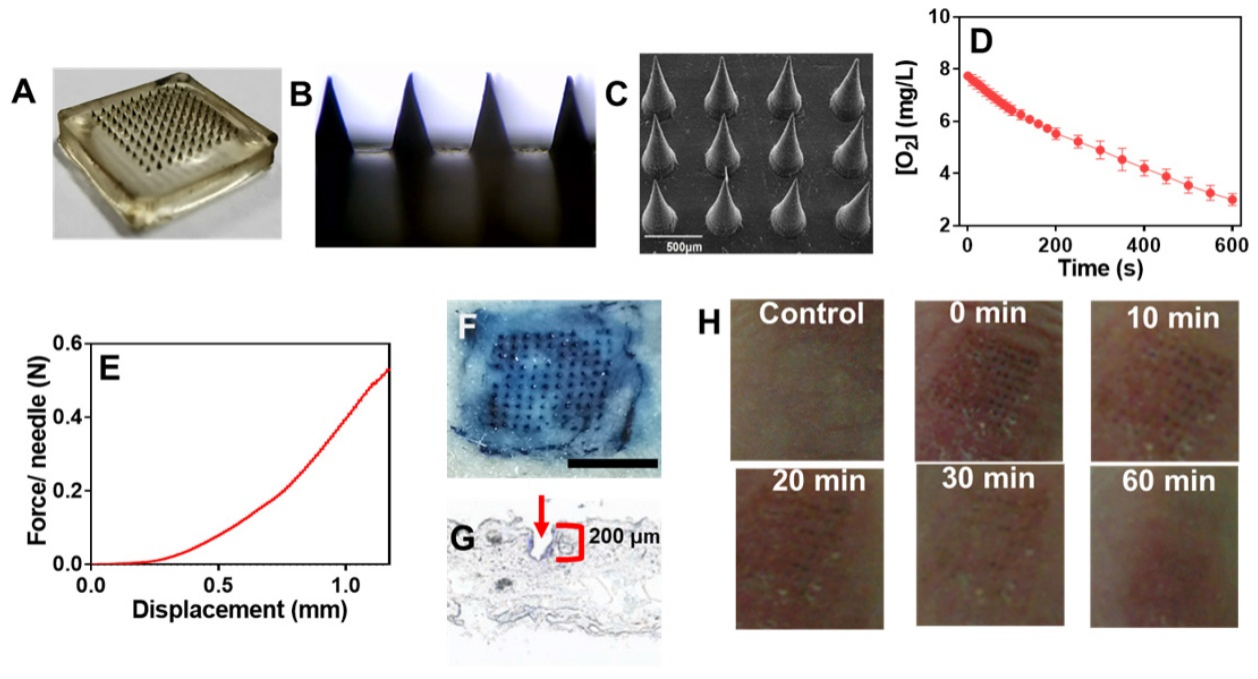
(A) High-resolution digital image and (B) micrograph of GOx@PDA MNs. (C) SEM image of GOx@PDA MNs in drying state, (Scale bar: 500 μm). (D) Rate of O_2_ consumption to measure the GOx@PDA loading into MNs. The assay was performed in presence of 2.5 mg/mL glucose. (E) Mechanical property of the MN. The failure force for desired MN was quantitatively measured as 0.58 N/needle. (F) Trypan blue staining showing MN penetration into mouse skin. Scale bar: 1 mm. (G) Image of frozen tissue sections to observe the cross-sectional area of mouse skin, which indicates penetration depth of MNs. The arrow indicates the insertion site. (G) The appearance of the skin before and after treatment with MNs at different timepoints.

Having confirmed the anti-tumor activity, we next aimed to incorporate the GOx@PDA into MNs for *in vivo* administration. MNs were prepared by using hyaluronic acid (HA) and polyvinylpyrrolidone (PVP) as matrixes owing to their excellent biocompatibility, mechanical property, and tailored cross-linking density 35, 47. Using the commercially available micro-molding (Figure 4A), MNs were fabricated with uniformed size and morphology (Figure 4B). From SEM characterization, an array of 100 MNs was assembled on a 2 × 2 cm^2^ patch with center-to-center interval of 600 μm (Figure 4C), and the GOx@PDA was concentrated at the tip of the needles through multiple deposition. Each needle was of a conical shape, with 500 μm in diameter at the base, 600 μm in height, and a sharp tip tapering to a 5 μm radius of curvature, which were exactly in accordance with the master mold. Compared with the control MNs (Figure S14), GOx@PDA-loaded MNs appeared to be black color in the needle tips, indicating that GOx@PDA was concentrated on tips of the pyramid in the MN. To quantify the GOx@PDA loading, the MNs were dissolved and the GOx@PDA was collected to measure the enzymatic activity (Figure 4D), based on which each MN was calculated to contain 700 μg GOx@PDA. The prepared MNs exhibited excellent stability in a silica gel environment for at least 15 d (Figure S15A), and the loaded GOx largely maintained its catalytic activity (Figure S15B).

Compared with systematic administration, topical drug delivery is compared favorably owing to its merits of increased patient compliance, skin targeting, and minimized side effects, etc 48, 49. However, the stratum corneum presents the main barrier for transdermal drug delivery, which hinders the penetration of most drugs across the skin for effective adsorption 50. MNs can elegantly solve this issue via non-invasively puncturing the stratum corneum, while mechanical strength is the key parameter for MNs to pierce stratum corneum. To characterize this, the mechanical compression test was performed (Figure 4E). The force acting on MNs continuously enhanced with the increase of the displacement of MNs, and the force reached 0.58 N/needle with the displacement value of 1.2 mm, providing sufficient strength for skin insertion without needle breaking 47. To further examine the strength of MNs, *in vitro* insertion test was carried out using the isolated abdominal skin of porcine, from which the skin insertion ratio and insertion depth were evaluated. The 100-needle arrays can be easily inserted into the pig cadaver skin using the gentle force of a thumb, and the insertion ratio was calculated to be 95% based on the trypan blue staining (Figure 4F). From the histological examination, the penetration depth was 150-200 μm (Figure 4G), indicating the successful penetration of the skin across stratum corneum and viable epidermis into the superficial dermis. Note that the MNs were ~ 600 μm in length, while they only reached the dermis layer without piercing through the skin. This might be ascribed to the elasticity of the skin, as well as the rapid dissolve of MNs matrixes during the insertion process.

To conform the percutaneous delivery of GOx, the penetration of GOx was examined by the Franz diffusion cell. The MNs were inserted into the isolated abdominal skin of porcine for 0.5 h incubation, and the receiving solution was sampled and centrifuged to collect the GOx@PDA. Then, the concentration of GOx@PDA was determined by the catalytic activity (Figure S16). Based on the rate of O_2_ consumption, the penetrated GOx@PDA was estimated to be 70%. The receiving solution was further centrifuged to collect the penetrated nanoparticles (Figure S17), and only slight size increase was observed, indicating high stability (Figure S18). Therefore, the MNs could easily pierce the stratum corneum and dissolve quickly to release GOx@PDA for the transdermal drug delivery.

We next explored the biocompatibility of the MNs by evaluating the skin recovery after treatment. The MN patch was applied to the back skin of C57 nude mice, and withdrawn 2 min later. At 30 min post insertion, the majority of erythema faded, and microchannels created by MNs were almost resealed (Figure 4H), thus avoiding the risk of infection and entry of pathogens. After 1 h, the skin almost recovered to the original condition, and no pathological change was observed after treatment based on H&E staining (Figure S19). Therefore, the MNs were minimal invasive to skin owing to the small size of needle tip as well as the fast-dissolving of the matrixes, making the MNs highly compliant for patient administration.

### *In vivo* Tumor ablation

Finally, the *in vivo* tumor ablation activity of GOx@PDA was evaluated by transdermal delivery using MNs patch. The tumor bearing mice model was built by subcutaneous implantation of B16F10 in the rear dorsal area of female C57BL/6 mice. When the tumor size reached ~ 50 mm^3^, the mice were divided into four groups for different treatments via local administration onto the tumor site. The mice without any treatment were used as control. For different formulations, MNs patch indicated blank MNs without any drug loading, while GOx/PDA MN meant the MNs loading with physical mixture of GOx and PDA shell. By virtue of the photothermal capability of GOx@PDA, transdermal delivery and tumor penetration of the nanosystem was dynamically monitored by using an infrared thermal imaging camera under laser irradiation. Compared to the control MNs, remarkable temperature increase was observed at 0.5 h after topical administration of GOx@PDA (Figure S20), while the photothermal signal was weakened overtime, indicating gradual penetration of GOx@PDA into tumor tissue.

**Figure 5:**
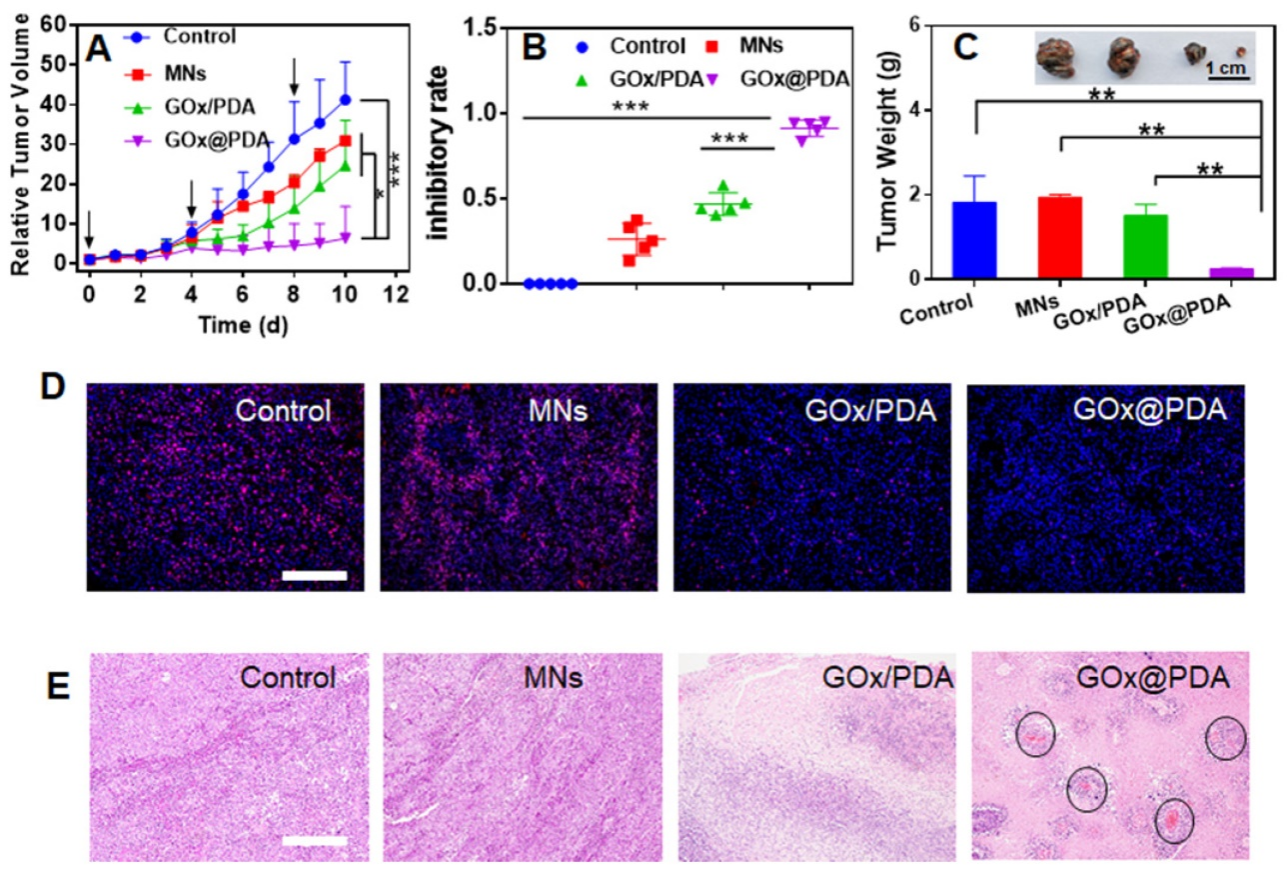
(A) Dynamic monitoring the tumor growth after different treatments. (B) tumor growth inhibitory rates of B16F10 tumor-bearing mice after different treatments. (C) Weight of excised tumor tissues after different treatments. Inset: the photograph of the excised tumor tissues. (D) Ki-67 staining and (E) H&E staining of the tumor tissue. For H&E staining, the black circle indicates angiogenesis. Scale bar: 100 μm.

The therapeutic efficacy was monitored every day by measuring the tumor volume. Without any treatment, the tumor grew quickly, resulting in 40-fold growth in 10 days (Figure 5A). The blank MNs have little effect on tumor growth, likely due to the mechanistic disruption of the tumor tissue. For the MNs loading with physical mixture of GOx and PDA, initial suppression was observed, while tumor started to re-grow at day 6, attributable to rapid deactivation of GOx *in vivo* by various biological ligands and proteinase. Specifically, most notable tumor growth inhibition was achieved for GOx@PDA MNs, resulting in the highest inhibition ratio of 91% (Figure 5B). The better efficacy for GOx@PDA MNs can be ascribed to the enzyme protection effect, enabling long-last catalytic consumption of glucose for effective starvation therapy.

For direct comparison, the tumor was extracted from mice body, and significant tumor shrinkage was observed for GOx@PDA MNs (Figure 5C, Figure S21), highly consistent with the *in vivo* observation. To further check the therapeutic efficacy, the tumor slices were collected for immunofluorescence staining and histological examination (Figure 5D-E). Based on Ki-67 staining, proliferative cells markedly decreased after treatments with GOx loading MNs (Figure 5D, Figure S22), confirming the mechanism of starvation therapy to inhibit cell proliferation. In addition, significant cell nuclear necrosis, vacuoles, and irregular widened interstitial space of the tumor tissue were seen from H&E staining (Figure 5E). Interestingly, we also observed angiogenesis after GOx@PDA therapy, likely due to the response of tumor towards GOx-induced starvation and tumor hypoxia 51. Through neovascularization, tumor tissue receives extra oxygen and energy supplies to resist starvation therapy, which may explain incomplete tumor eradication after GOx@PDA MNs treatment. It also indicates a combination of GOx@PDA with antiangiogenic drugs for synergistic tumor therapy in the future. Over the treatment period, no obvious side effects were observed, and the body weights of all mice slightly increased (Figure S23). Notably, compared with intravenous injection that caused an obvious blood glucose decrease, no fluctuation of blood glucose level was observed for GOx@PDA administrated via MNs (Figure S24). It is known that the PDA nano-shell could be finally degraded by biological ligands such as GSH and tumor acidic environment, followed by elimination form body 52. We finally tested the systematic compatibility by H&E staining (Figure S25), and none of the organ showed any pathological changes after treatment. All these results demonstrated the merits of MNs-based transdermal drug delivery system for enhanced biosafety.

## Materials and Methods

### Materials

Polydimethylsiloxane (PDMS) were purchased from Dow Corning Corp. (Midland, MI, USA). Hyaluronic acid (HA, MW = 170 kDa), polyvinyl alcohol (PVA, MW = 44.5 kDa), polyvinyl pyrrolidone (PVP, MW = 100 kDa), folic Acid (FA), glucose Oxidase (GOx), dopamine (DA), protease K, and 3-(4, 5-dimethylthiazol-2-yl)-2,5-diphenyl tetrazolium bromide (MTT), ZnCl_2_ were purchased from Sigma-Aldrich (Saint Louis, MO). Disodium hydrogen phosphate (Na_2_HPO_4_), and sodium dihydrogen phosphate (NaH_2_PO_4_) of reagent grade quality were obtained from Hong shun (Shanghai, China). 8-hydroxy-5-quinolinesulfonic acid (HQS) was from Sigma-Aldrich (Saint Louis, MO), and 4-(2-hydroxyethyl)- piperazine-1-ethanesulfonate (HEPES) was from Mandel Scientific (Guelph, Ontario, Canada). Silica gel, glucose and dimethyl sulfoxide (DMSO) were purchased from Sinopharm Chemical Reagent Co., Ltd (Shanghai, China). BCA protein quantification kit was obtained from Beijing Dingguo Changsheng Biotech Co., Ltd (Beijing, China). Trypan Blue and Tris (hydroxymethyl) aminomethane (Tris) were purchased from Soleibao Technology Co., Ltd (Beijing, China). Dulbecco's Modified Eagle Medium (DMEM), Roswell park memorial institute 1640 medium (RPMI-1640), 3-(4,5- dimethylthiazol-2-yl)-2,5-diphenyltetrazolium bromide (MTT), penicillin-streptomycin solution, and fetal bovine serum (FBS) were purchased from Gibco Life Technologies (Gaithersburg, MD, USA). Live/dead viability/cytotoxicity assay kit was obtained from Nanjing KeyGEN Biotech. Co., Ltd. (Jiangsu, China). The 2′,7′-dichlorodihydrofluorescein diacetate (DCFH-DA) probe was obtained from Macklin Co., Ltd (Shanghai, China). ATP assay kit was purchased from Beyotime Institute of Biotechnology (Shanghai, China). B16F10, 4T1, A549 and MDA-MB-231 cells were obtained from Xiangya cell center (Changsha, China).

### Preparation and characterizations of GOx@PDA

FA (2 mL, 2 mM), GOx (50 μL, 0.5 mg) and PVA (1 mL, 2%) were dissolved in 0.5 mL ultrapure water, and 0.5 mL ZnCl_2_ (8 mM) was added under vigorous stirring, followed by sonication for 5 min. Then, 400 μL Tris (0.1 M, pH 8.0) was added to adjust the pH to 7.6, and DA (5 mg/mL) was added for 48 h self-polymerization. NCPs/GOx@PDA was collected via centrifugation, and dialyzed by phosphate-buffered saline (pH 7.4, 10 mM) for 1 h to etch the NCPs core. The resulting GOx@PDA was obtained by centrifugation, and dispersed in water for further use. Morphology of the nanoparticles was observed by transmission electron microscopy (TEM, Titan G2 60-300, FEI) at 200 kV. ζ-Potential, size, and size distribution of were measured with a dynamic laser scattering (DLS, Nano ZS, Malvern Instruments, U.K.). UV-vis absorption measurement was performed on a UV-vis spectrophotometer (UV2450, Shimadzu Corp.). The loading amount of GOx was analyzed by bicinchoninic acid (BCA) protein assay (Beyotime).

### Quantification of Zn^2+^ and FA

Various concentrations of Zn^2+^ were mixed with 2 mM HQS (in HEPES buffer, 10 mM, pH 7.5), and the fluorescence was measured by plate reader (Ex = 393 nm, Em = 521 nm) to build the standard curve for Zn^2+^ quantification. Likewise, the standard curve for FA was built by dissolving folic acid in HEPES buffer (pH 7.4 10 mM) with a series of concentrations, which were measured by using high performance liquid chromatography (HPLC). To extract Zn^2+^ and FA, the nanoparticles were treated with 20 mM GSH for 2 h, and the supernatants were collected by centrifugation (16000 rpm, 10 min) for subsequent quantification using the standard curves as described above.

### Zn^2+^ release study

The Zn^2+^ release was assessed by dialysis. NCPs/GOx@PDA were dissolved in 10 mM phosphate buffer solutions (pH 7.4). At predetermined intervals, sample was withdrawn and centrifuged, and Zn^2+^ concentration in supernatant was detected by HQS.

### Catalytic activity of GOx@PDA

GOx@PDA (3.5 mg/mL) was mixed with glucose (2.5 mg/mL), and the real-time pH change and oxygen consumption was monitored by a pH meter (PHS-3E, INESA Scientific Instrument Co., Ltd., China) and a dissolved oxygen meter (JPBJ-609L, INESA Scientific Instrument Co., Ltd., China), respectively. To explore the protection effect, free GOx or GOx@PDA (equal GOx concentration of 0.5 mg/mL) was mixed with cell lysate, 10% serum or phosphate-buffered saline (pH 7.4). After different incubation time periods at 25 °C, the catalytic activity was measured by monitoring the oxygen consumption. To investigate the protection ability against proteases, free GOx or GOx@PDA (equal GOx concentration of 0.5 mg/mL) was treated with proteinase K (5 mg/mL) at 37 °C for 2 h under gentle shaking. Then, the activity was measured by monitoring pH variation and oxygen decrease. Likewise, the enzymatic activity of free GOx or GOx@PDA under harsh conditions was also measured by treating the samples with phosphoric acid solution (pH 4.0), phosphoric acid solution (pH 5.5 and 6.5), or ammonia solution (pH 10).

### GOx release from GOx@PDA

The release of GOx was measured by a membraneless dissolution method. Typically, 200 μL GOx@PDA was mixed with 800 μL release medium under gentle shaken at 37 °C. Samples were taken at predetermined time points, and the concentration of GOx was analyzed to calculate cumulative drug release.

### Cell culture

B16F10 cells, A549 cells and MDA-MB-231 cells were incubated in DMEM medium containing 10% FBS and 1% antibiotics (penicillin-streptomycin, 10000 U/mL), while 4T1 cells were incubated in RMPI 1640 medium containing 10% FBS and 1% antibiotics (penicillin-streptomycin, 10000 U/mL). The cells were cultured at 37 °C in a humidified atmosphere containing 5% CO_2_.

### MTT assay

The cells were seeded into 96-well plates at a density of 5000 cells per well for 24 h culturing. Then, gradient concentrations of GOx@PDA were added for 48 h incubation, followed by adding MTT (100 μL, 0.5 mg/mL). After another 4 h incubation, the media were replaced with 100 μL of DMSO, and the absorbance was measured using a microplate reader (Infinite M200 Pro, Tecan, Morrisville, NC, USA). The cell viabilities (%) were calculated as follows: 100 × OD (samples) / OD (control), where OD (control) and OD (samples) represent the absorbance at 490 nm for the cells with different samples treatments and non-treatment control, respectively.

### Intracellular ATP quantification

B16F10 cells were seeded into a 6-well plate with an initial cell density of 2 × 10^4^ cells/cm^2^. When the cell confluence reached about 70%, the cells were incubated with PBS (control), PDA shell (4 μg/mL), or GOx@PDA (4 μg/mL) for 24 h, respectively. Subsequently, the cells were collected and counted to ensure that each sample contained the same number of cells. The intracellular ATP content was measured by following the user manual of the ATP assay kit.

### Reactive oxygen species probing

The B16F10 cells were seeded onto 24-well plates at density of 4 × 10^4^ cells per well. After 48 h, the cells were treated with PDA shell or GOx@PDA for 6 h. Then, 10 μM of DCFH-DA was added for 30 min incubation. The cells were then imaged by fluorescent microscopy.

### Cell Live/Dead staining

B16F10 cells were seeded into the 35 mm × 12 mm CLSM cell culture dish (NEST Biotechnology Co., Ltd., China). When the cell confluence reached about 70%, the original culture medium was replaced by a fresh one containing PBS (control), PDA shell (4 μg/mL), or GOx@PDA (4 μg/mL), followed by 48 h incubation. Then, the cells were stained with Calcein AM/Propidium Iodide (PI) for 15 min and analyzed using a confocal microscope (LSM 510 Metanlo, Zeiss Co., Germany).

### Preparation and characterizations of the MNs

To prepare the blank MNs, PVP K30 (40%) and HA (15%) were poured into the PDMS mold under vacuum, and the sample was dried in a sealed desiccator overnight, followed by peeling off from the molds, and stored in a desiccator. To prepare drug-loaded MNs, GOx@PDA solution was first covered and filled into a microneedle mold under vacuum. Thereafter, redundant solution was removed to make sure each cavity was filled with the same volume of drug solution. Then, the solution was evaporated for a short period to concentrate GOx@PDA on MN tips before filling the PVP K30 and HA matrixes to form the base. Morphologies of MNs were observed by a scanning electron microscope (Siron 200, FEI, Eindhoven, The Netherlands). To determine the drug loading, MNs were dissolved in a certain volume of phosphate buffer, and GOx were quantified by measuring its catalytic activity.

The mechanical strength was measured using a tensile load frame. The tensile force was continuously monitored as a stainless-steel plate compressing arrays of microneedles along the y-direction on a stress-strain gauge. The initial gauge was set at 2.00 mm between the MN tips and the stainless-steel plate, the speed of the top stainless steel plate movement towards the MN-array patch was 0.1 mm/s. The failure force of MNs was recorded as the needle began to buckle.

To assess the skin insertion capability, MNs were applied onto the dorsal skin of a mouse for 5 min. Then, the mouse skin was retrieved and stained with 0.4% trypan blue solution for 1 h, followed by washing with water. Then the skin was observed under an AxioCam MRc5 microscope (Carl Zeiss Microscopy GmbH, Oberkochen, Germany), and pictures were taken with a high-definition mobile phone (Huawei P30). The insertion ratio is equal to the number of dots on the skin surface divided by the number of needle tips on each microneedle. The depth of the microneedles inserted into the skin was measured on frozen tissue sections.

The skin penetration of the MNs were evaluated by using Franz transdermal diffusion cell. Briefly, the MNs were applied to depilated back skin via thumb insertion for 5 min, and then the skin was transferred to Franz transdermal diffusion cell (the transdermal area was 2.50 ± 0.08 cm^2^) with receiving cell containing 2.1 mL phosphate-buffered saline solution. After 0.5 h incubation (300 rpm, 32 °C), the receiving solution was collected to quantify the GOx@PDA penetration.

The recovery of the skin after MNs treatment was studied on the back skin of C57 mice. The MNs were inserted into skin using thumb and index finger for 5 min. After removing the MNs, the morphological changes of the skin were monitored at different timepoints.

### Photothermal imaging in vivo

The in vivo photothermal images were pictured using an FLIR C2 camera at 0.5 h post-injection of GOx@PDA MNs laser irradiation at a power density of 0.9 W/cm^2^ for 1 min.

### *In Vivo* Antitumor Activity

C57 (4 to 5 weeks old) were obtained from Changzhou Cavens Lab animal Co., Ltd. (Jiangsu, China). The animals were maintained in a sterile environment and allowed free access to food and water. All animal experiments were approved by the Experimental Animal Ethics Committee of Central South University and carried out in accordance with the requirements of the National Act on the use of Experimental Animals (people's Republic of China). The mice were seeded with B16F10 cells at a density of 1 × 10^6^ tumor cells to establish the subcutaneous melanoma mice model. When the tumors reached ~50 mm^3^ in diameter (7-10 days after injecting the cells), the mice were randomized into four groups: (1) control; (2) blank MNs without GOx loading; (3) GOx/PDA MNs loading of physical mixture of GOx and PDA shell; (4) GOx@PDA MNs. Each MN contained 105 μg GOx. All the groups received treatments on day 0, 3, 6. Body weights and tumor volumes (0.5 × length × width^2^) of the mice were recorded every day. The mice were sacrificed on day 10, and the tumors as well as the main organs were harvested for subsequent analyses, including weighting, hematoxylin & eosin (H&E) staining, and Ki67 immunofluorescent staining.

For photothermal imaging, the mice were pictured using an FLIR C2 camera at 0.5 h post-injection of GOx@PDA MNs after laser irradiation (0.9 W/cm^2^ for 1 min).

### Blood glucose measurement

Healthy mice were randomized into three groups: (1) control (no treatment); (2) intravenous injection of GOx@PDA NPs (100 μL, 7 mg/mL); (3) topical administration of GOx@PDA MN (700 μg MNs). At pre-determined timepoints, 25 μL blood sample was taken from the tail vein, and the blood glucose concentration was measured by a blood glucose meter.

### Statistical analysis

All quantitative data were expressed as mean ± SD from at least triplicate measurements. Differences between two comparative groups were assessed using the Student's t-test, and the significance among multiple groups was examined by the one-way analysis of variance (ANOVA). Significance was measured at the following thresholds: *P < 0.05, **P < 0.01, ***P < 0.001, ****P < 0.0001.

## Conclusions

In summary, we designed and fabricated a PDA-based nano-capsule to load GOx for long-lasting catalytic starvation therapy of melanoma via MNs-based transdermal delivery. FA/Zn^2+^-coordinated NCPs were employed as templet for GOx encapsulation and self-polymerization of PDA shell layer, and then NCPs nano-core was etched to improve the biocompatibility of the nanosystem. The resulting GOx@PDA can retain its catalytic activity for at least 6 d in presence of biological matrixes including serum and cell lysate, and resist various harsh conditions (acid/base treatments, and proteinase-induced degradation), all of which were benefited from the protective effect of PDA nano-capsule. In vitro, GOx@PDA showed excellent anti-tumor effect against various types of tumor cells via catalytic consumption of glucose to cut off the energy supply. For *in vivo* application, GOx@PDA was integrated into dissolving MNs, and the MNs were well-characterized in terms of micro-structure, drug loading, skin penetration, and biocompatibility. Upon transdermal administration, significant tumor growth inhibition was achieved without any noticeable side effects, while accompanied by angiogenesis in tumor tissue. Therefore, it shows the potential of combining GOx@PDA with antiangiogenic drugs for better therapeutic outcomes. This work addresses the key limitations of GOx for *in vivo* applications, and pointing out the future direction of GOx-based starvation tumor therapy for enhanced efficacy.

## Supplementary Material

Supplementary figures.
