# Supplementary material for: Cell membrane inspired nano-shell enabling long-acting Glucose Oxidase for Melanoma starvation therapy via microneedles-based percutaneous delivery
Source: Theranostics. 2021 Jul 13;11(17):8270–82. doi: 10.7150/thno.60758 (PMC8344000; doi:10.7150/thno.60758)
Supplement: Supplementary file 1 — Supplementary figures. [file thnov11p8270s1.pdf]

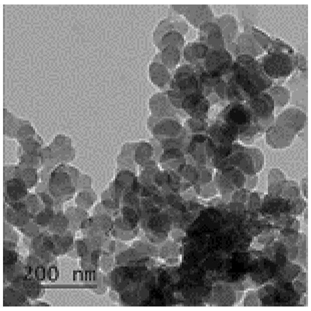

Figure S1. The TEM image of NCPs/GOx.

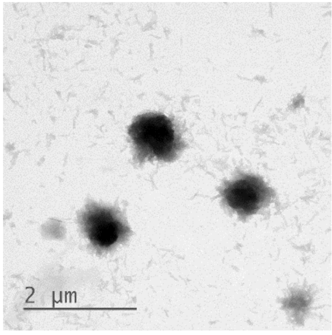

Figure S2. The TEM image of GOx@PDA.

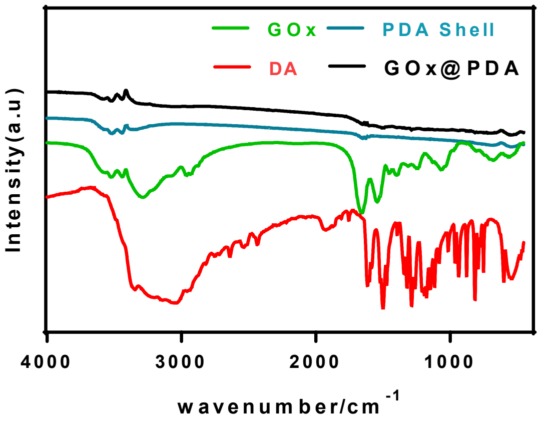

Figure S3. FT-IR spectra of GOx, DA, PDA shell and GOx@PDA.

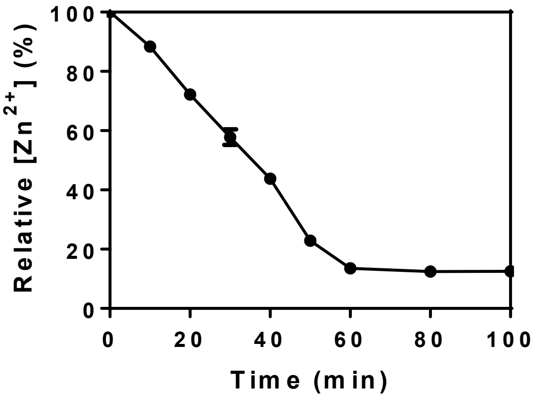

Figure S4. The relative content of Zn^{2+} in NCPs/GOx@PDA after different etching time periods.

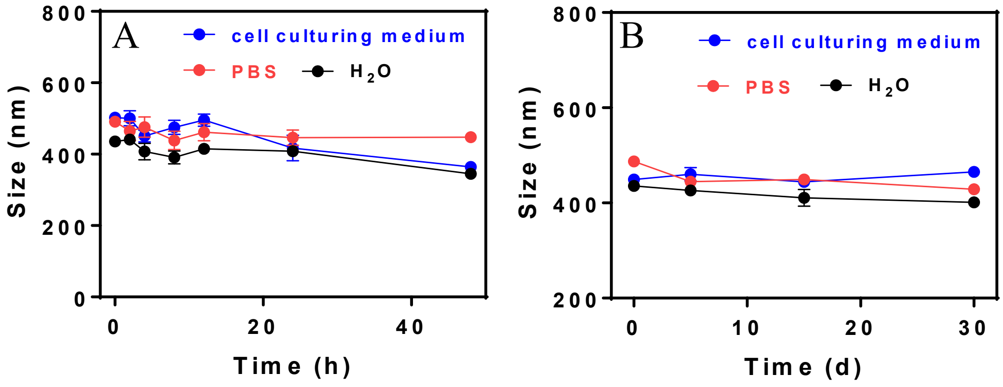

Figure S5. (A) Dynamic particle size change of GOx@PDA in water, PBS (pH 7.4), and cell culturing medium at 37 ° C for 48 h incubation. (B) The long-term colloidal stability of GOx@PDA at 4 ° C.

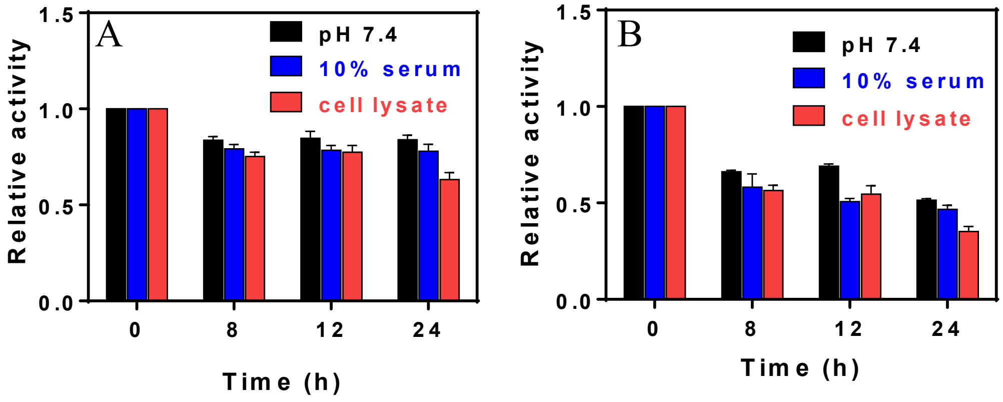

Figure S6. (A) Relative catalytic activity of (A) GOx@PDA and (B) free GOx after incubation with PBS buffer (pH 7.4), 10% serum and cell lysate obtained from B16F10 melanoma cells for different time periods at 37 ° C.

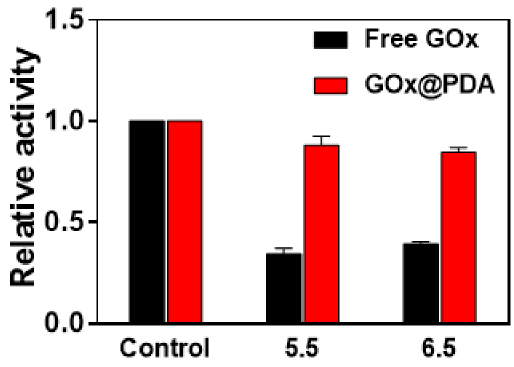

Figure S7. Relative activity of GOx@PDA and free GOx after treatments with phosphate buffers (pH 5.5 and pH 6.5) for 10 min.

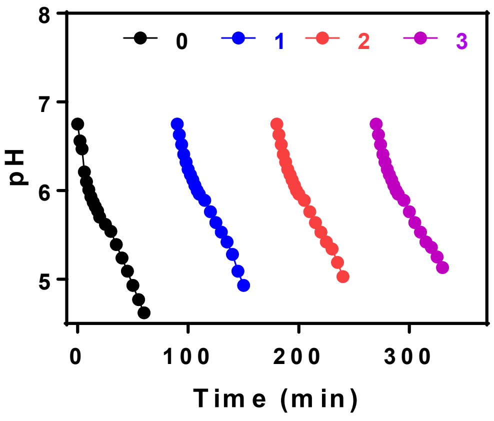

Figure S8. Catalytic activity of GOx@PDA after various rounds of treatment with proteinase by monitoring the pH decrease.

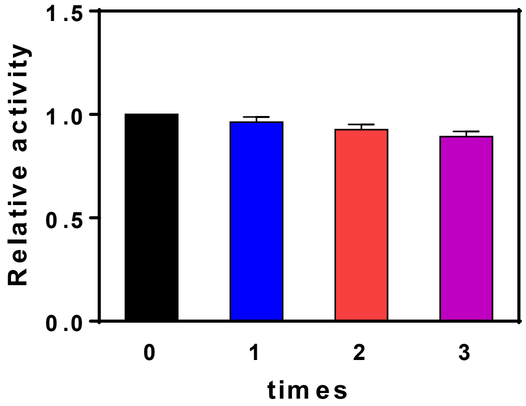

Figure S9. Catalytic activity of GOx@PDA after various rounds of treatment with proteinase by monitoring oxygen consumption.

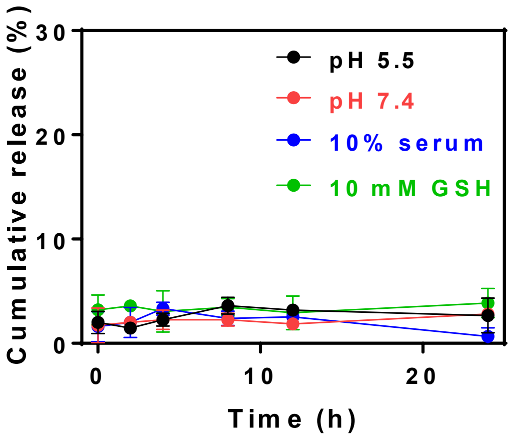

Figure S10. The release GOx from GOx@PDA under different conditions.

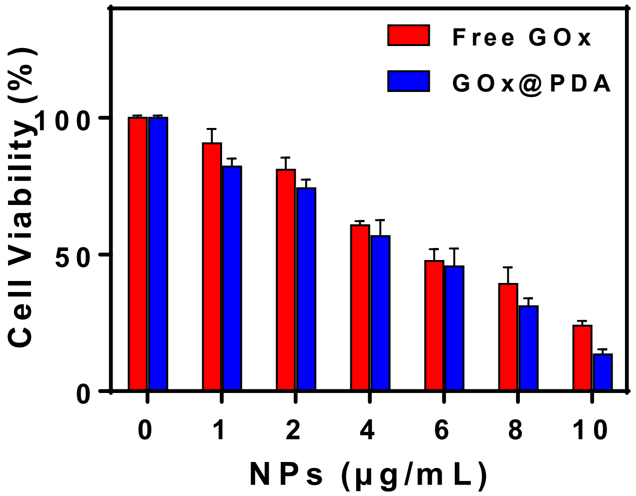

Figure S11. Cell activity of B16F10 cells after treatment with free GOx and GOx@PDA at different concentrations for 48 h.

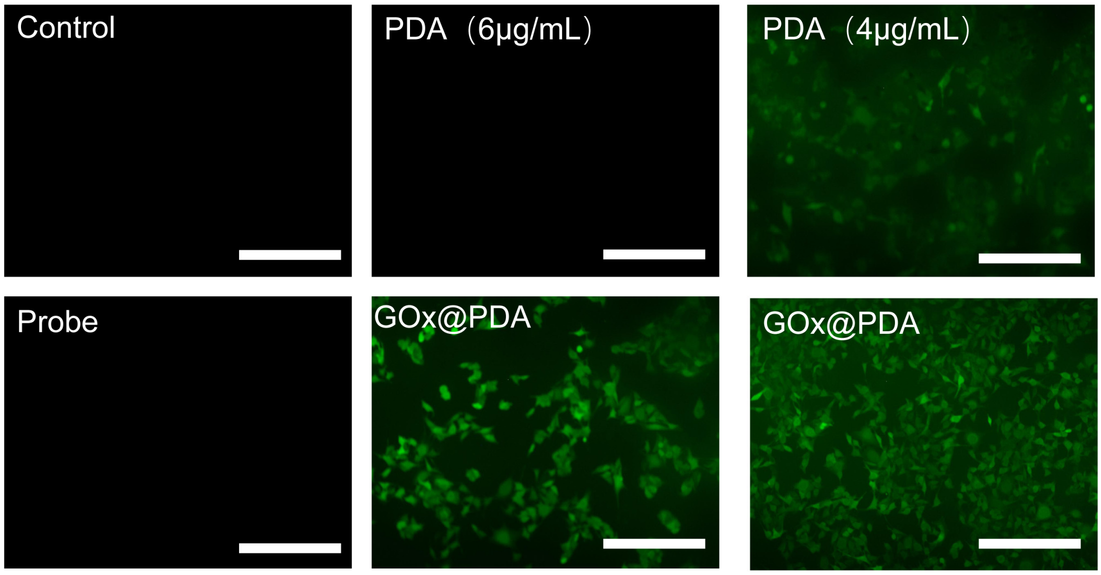

Figure S12. Fluorescent images of B16F10 cells treated with PDA shell and GOx@PDA for 6 h and then incubated with DCFH-DA for 30 min. Scale bars, 100 μm.

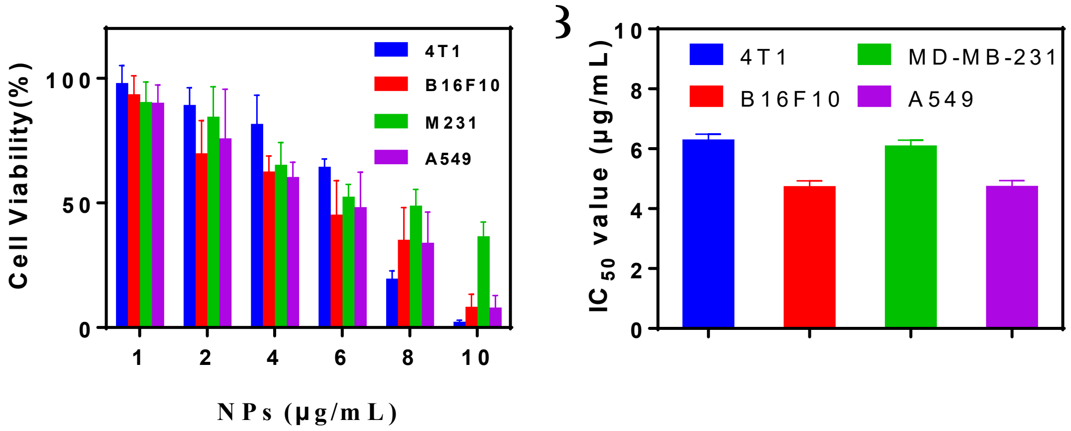

Figure S13. (A) Cell viability and (B) IC_{50} values of GOx@PDA for various types of tumor cells after 48 h incubation.

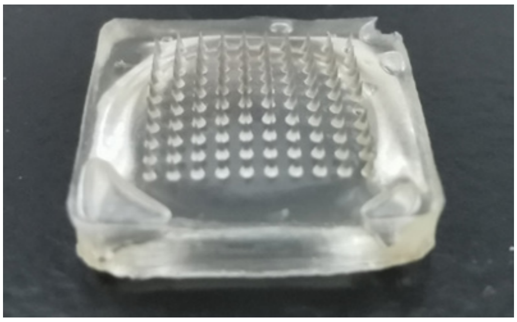

Figure S14. High-resolution digital image of the blank MNs

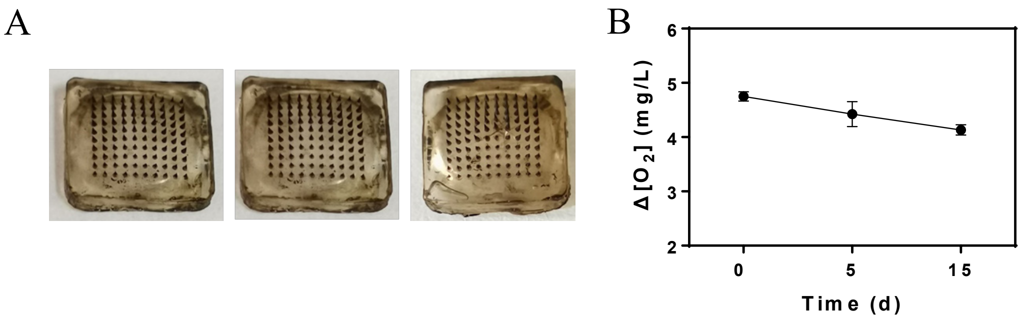

Figure S15. (A) The stability of MNs in a silica gel environment for 15 d storage. (B) The catalytic activity of GOx in MNs for 15 d storage.

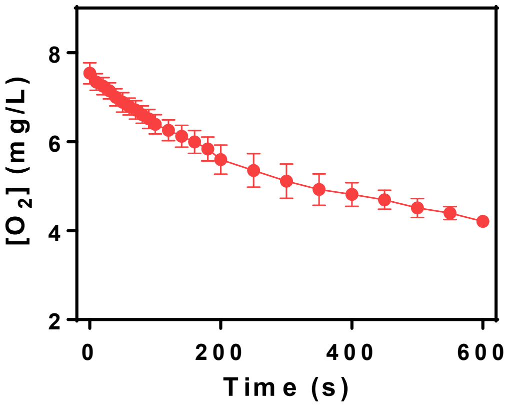

Figure S16. Quantification of the skin penetrated GOx@PDA by measuring the catalytic activity of GOx.

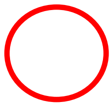

Figure S17. The nanoparticles in receiving solution were collected by centrifugation to demonstrate their successful penetration.

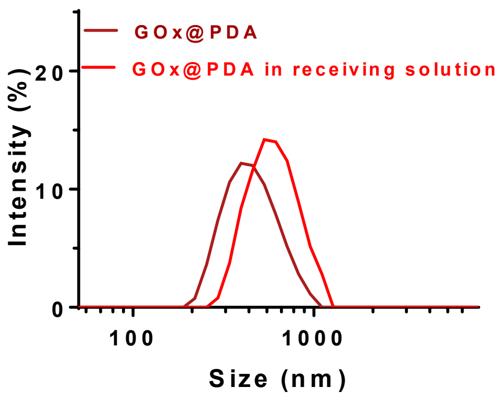

Figure S18. DLS measuring the size of GOx@PDA before and after penetration into skin.

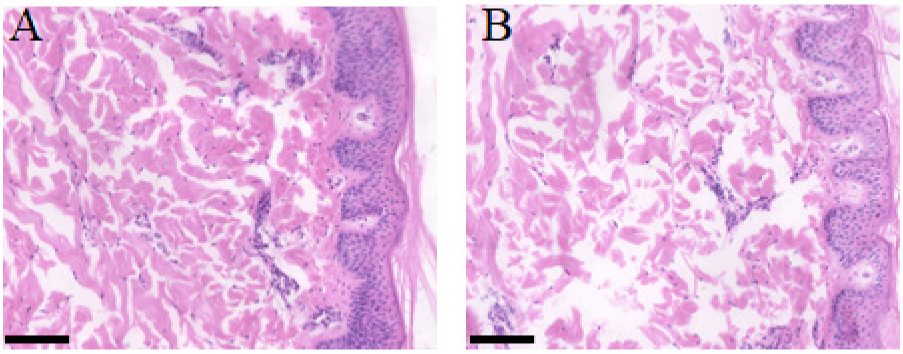

Figure S19. H&E staining of mouse skin without (A) or with (B) treatment of GOx@PDA MNs.

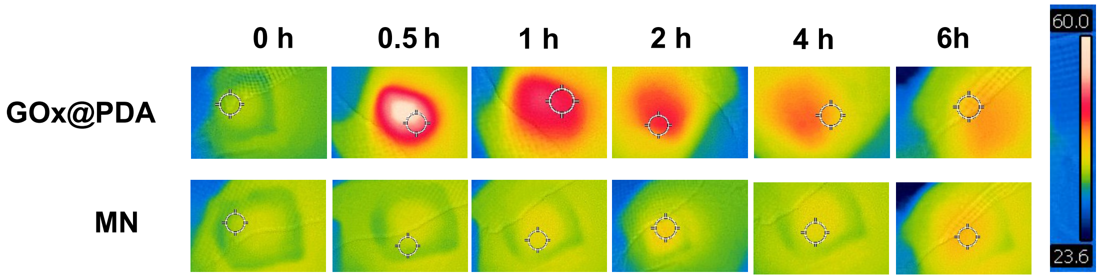

Figure S20. Photothermal imaging of mice treated with GOx@PDA MNs and blank MNs after laser irradiation.

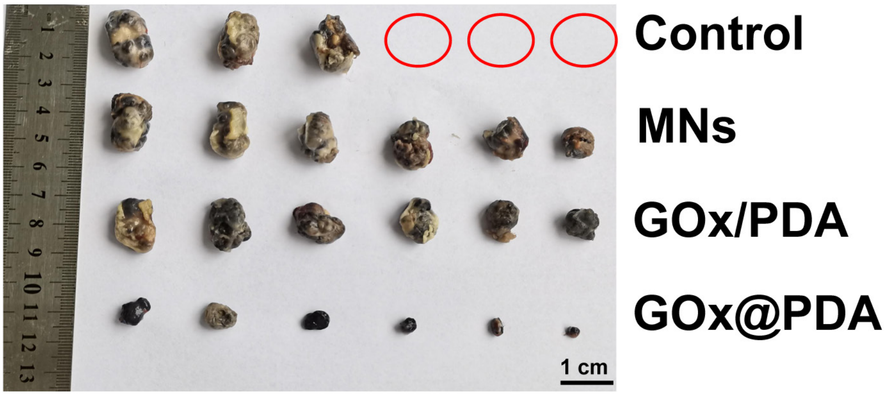

Figure S21. Photographs of the harvested tumors after different treatments, and the blank space indicates complete tumor eradication.

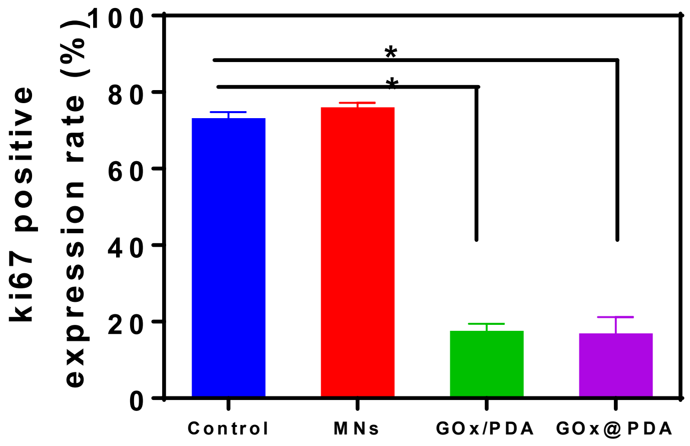

Figure S22. Quantified Ki67 immunofluorescent intensity after different treatments.

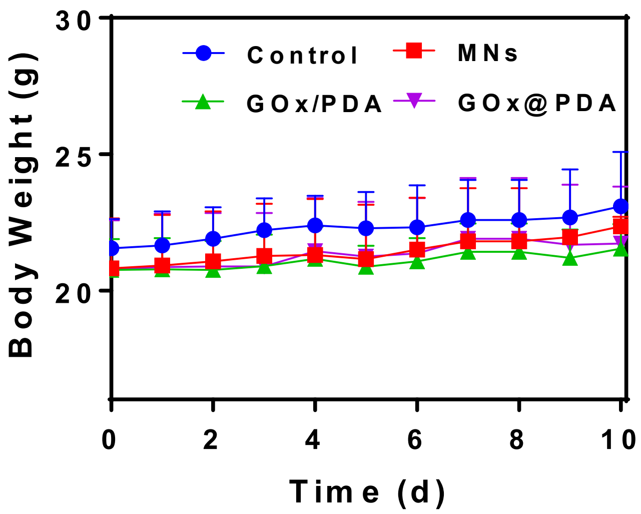

Figure S23. Dynamic monitoring the body weight of the mice during different treatments.

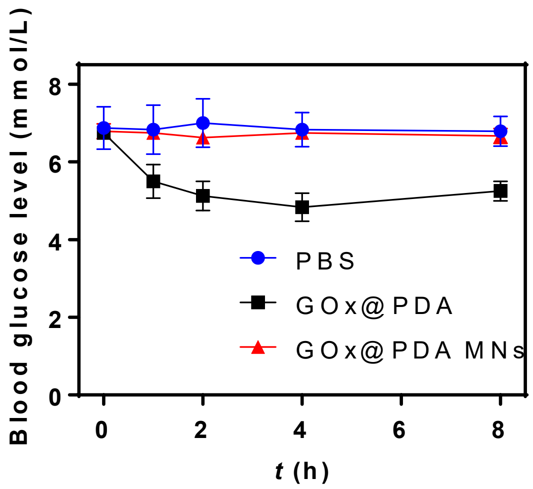

Figure S24. Dynamic monitoring the blood glucose level after different treatments. GOx@PDA were administrated via intravenous injection, while GOx@PDA MNs were topically delivered.

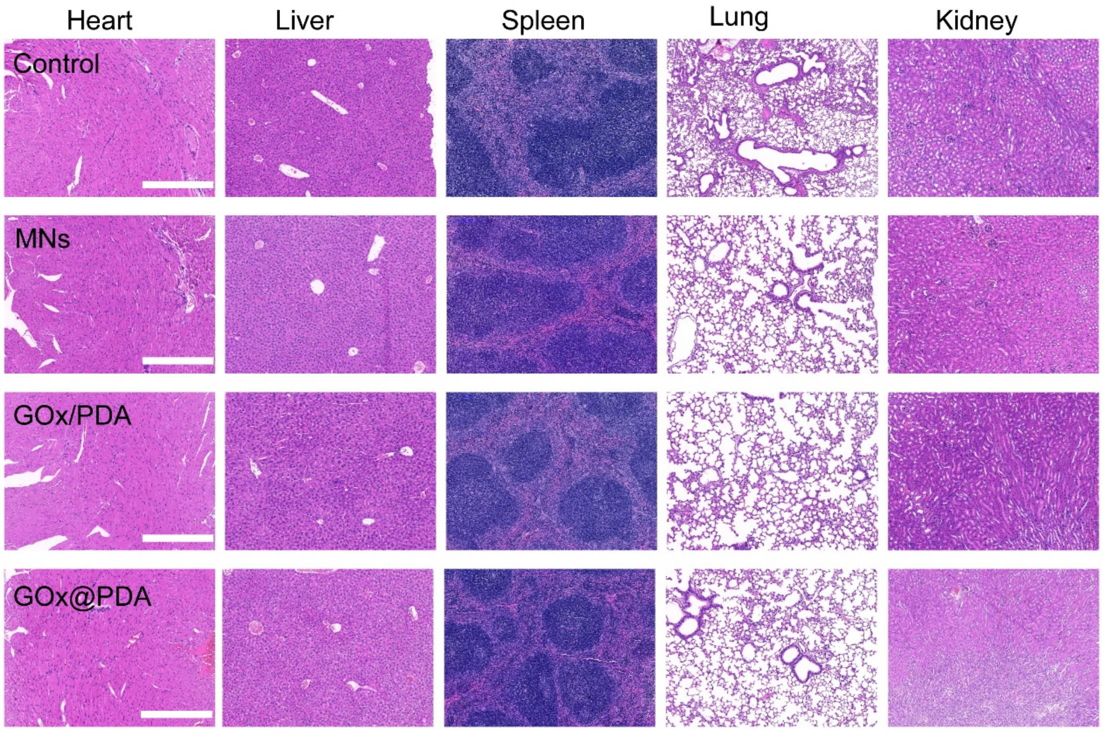

Figure S25. H&E staining of the main organs from mice after different treatments.
